# Applying the nominal group technique to determine emerging stressors related to youth mental health: findings from a multi-country stakeholder consensus-building exercise

**DOI:** 10.3389/fpsyt.2025.1651933

**Published:** 2025-11-07

**Authors:** Nazeema Isaacs, Zaynab Essack, Lilian Mutengu, Salome Wawire, Susan Gichoga, Alphonsus Neba, Uzma Alam, Constance Mabia, Palesa Sekhejane, Byron Bitanihirwe

**Affiliations:** 1 Impact Centre, Human Sciences Research Council, Cape Town, South Africa; 2 Public Health, Societies, and Belonging, Centre for Community Based Research, Human Sciences Research Council, Pietermaritzburg, South Africa; 3 School of Law, University of KwaZulu-Natal, Pietermaritzburg, South Africa; 4 The Science for Africa Foundation, Nairobi, Kenya; 5 Walter Sisulu University, Eastern Cape, South Africa

**Keywords:** consensus, low- and middle-income countries, mental health, nominal group technique, relation wellbeing model, stressors, youth

## Abstract

**Backround:**

Youth represents a distinct phase of neurodevelopment encapsulating a unique mix of personal, social, and environmental stressors that can impact mental health and increase vulnerability to mental illness. To gain a cross-national understanding of the stressors that may impact young people’s mental wellbeing, we conducted a consensus-building exercise focused on ranking a list of stressors that emerged through stakeholder deliberation.

**Methods:**

We adopted the nominal group technique (NGT) as an exercise to reach a consensus among representatives from 11 low- and middle-income countries (spanning Africa, Asia, Europe, and Latin America) in terms of stressors linked to young people’s mental wellbeing. A single session of NGT was applied to probe what country representatives felt were the most pressing stressors associated with youth mental health in the context of the relational wellbeing model (at the personal, social, and environmental levels).

**Results:**

Representatives identified 18 stressors—that included mental health awareness, media, stigma, climate change and policy, among others—as being high priority for developing research geared towards youth mental health.

**Conclusion:**

There was a high level of consensus in terms of the stressors that were identified in relation to youth mental health, suggesting that use of NGT provides an effective tool to generate pertinent data from a single session with important research and policy implications. These findings underscore the need for more empirical research focused on knowledge gaps associated with the identified stressors—in terms of youth mental health—which can then better inform funding agendas as well as mental health policy and practice.

## Introduction

Youth is a distinct phase of life involving a complex journey of transition from childhood to adulthood that encapsulates a mix of personal, social, and environmental factors that ultimately serve to shape mental health (1, 2). The definition of youth is known to vary across cultures (3), but generally focuses on an individual’s developmental status in relation to cognitive, psychological, and economic markers that would classify them as having established adulthood (1, 2).

Adolescence represents an important inflection point on the journey of every person as they veer towards adulthood. Experiences during this critical stage of cognitive and psychosocial development impact mental health both positively and negatively and establishes a critical basis for vulnerability to mental illness (4). As our knowledge of young people and their mental health continues to grow, it is understood that a significant proportion of health problems among the young have at least a partial basis in mental health and/or substance use disorders (5). Indeed, it is estimated that almost 75% of all mental health challenges emerge before the age of 24 (6). The increased reported incidence of stressors in youth, including pressure to conform with peers, academic stress, and high rates of unemployment, paint a troubling picture of youth mental health (7).

There are three major categories that a stressor can be categorized into: psychological, physiological, and behavioral (8). Psychological stressors involve cognitive or emotional factors, such as worries and negative thoughts (e.g., social comparisons and self-esteem issues) that often manifest in the form of anxiety and depression (9). Physiological stressors include any physical stimuli that disrupt homeostasis such as metabolic abnormalities and infections (9). Behavioral stressors pertain to environmental or internal factors that lead to changes in behavior. These changes can manifest as maladaptive coping mechanisms, altered social interactions, or even substance use (8). For the present paper, we focus on psychological stressors experienced during youth.

During adolescence, individuals experience major personal changes such as the development of their self and identity as well as forming peer groups and developing close relationships with individuals inside and outside the family (10). This implies that adolescence may be a particularly stressful time. According to Zimmer-Gembeck and Skinner (10), approximately 25% of adolescents will experience at least one significant stressor related to school (e.g., being bullied by their peers, academic challenges, issues with teachers) and interpersonal relationships (e.g., conflict with parents or siblings and even peers) with potential detrimental effects on their wellbeing. The previous point is substantiated by work from Hellström and colleagues (11) who highlight that over the past thirty years, a substantial number of young people from Sweden reported having school-related stress and mental health problems. These researchers go on to suggest that the most common form of stressor experienced during adolescence is psychological. In their study, Hellström et al. (11) revealed that research conducted in this area mainly focused on high-income countries (HICs). To this end, Potrebny et al. (12) conducted a systematic review focusing on mental health complaints among seven million adolescents from 36 countries in Europe, North America, Israel, and New Zealand. The authors found a minor increase, albeit significant, in the prevalence of mental health complaints self-reported by adolescents. In yet another study, Hagquist et al. (13) enquired on adolescents’ psychosomatic symptoms from Finland, Denmark, Norway and Sweden and found increasing rates of mental health problems among this demographic. Unlike the rich and diverse research on youth mental health stemming from HICs, there remains a scarcity of research on this topic from low - and middle-income countries (LMICs), an aspect posing long term social and economic challenges (14).

In this context, the Being mental health initiative was conceived in 2022 to understand the drivers that impact the mental health of young people (aged 10 to 24), particularly in LMICs based on their own perspectives (7). This initiative focuses on preventive and promotive strategies to improve youth mental health and wellbeing via research, ecosystem engagement and innovation in 12 priority countries: Colombia, Ecuador, Ghana, India, Indonesia, Morocco, Pakistan, Romania, Senegal, Sierra Leone, Tanzania, and Vietnam. One crucial aspect of the Being initiative is gaining a better understanding of how stressors experienced during youth can impact subsequent mental health (7). To this end, a workshop was hosted in Nairobi, Kenya on 16–17 January 2024, involving mental health experts to identify key stressors impacting youth mental health across Being focal countries (15). A particular aspect of the event that required consideration involved appreciating the different definitions of “youth” in terms of the focal countries. For instance, while the African Youth Charter defines youth as those persons between the ages of 15 and 35, countries in Latin America and Asia as well as Romania define “youth” as people aged between 15 and 29.

Given the likely diversity of opinion that any group of people may display when considering a topic (i.e., stressors impacting youth mental health), formalized methods, such as consensus techniques, are essential for organizing subjective judgments in group work. Consensus techniques have been successfully used by several research groups in the mental health space, including developing frameworks for forensic mental health services (16) and exploring risks among young people in inpatient mental healthcare services (17). The two most common consensus methods used for medical and health research are the Delphi method and the nominal group technique (NGT) (18). The Delphi method is a forecasting method based on several rounds of questionnaires sent to a panel of experts. The anonymous, written responses are aggregated and shared with the group after each round (19). In contrast, the NGT is a structured, multistep, facilitated, group meeting technique used to generate and prioritize responses to a specific question by a group of people who have expert insight into a particular area of interest (16–18).

In this study, we describe the process of identifying key stressors impacting youth mental health in 11 focal LMICs via multi-country stakeholder consultation, using NGT. Specifically, the relational wellbeing framework model—where an individual’s inner wellbeing and subsequent psychological functioning is defined in terms of social, personal and environmental relationships (20) was used to divide the identified stressors into personal, societal, and environmental domains (21). The goal was to use the identified stressors to develop a funding call that was launched in February 2024.

## Research methods and design

### Representatives

Ten representatives (7 females and 3 males) of 11 LMICs (i.e., Senegal, Sierra Leone, Tanzania, Ghana, Ecuador, Colombia, India, Pakistan, Vietnam, Romania, and Indonesia) participated in the consensus-building process, with one individual representing both Senegal and Sierra Leone. All representatives in the group were adults aged 18 years or older, lived or worked in an LMIC, and conducted research related to youth mental health ranging from qualitative research and applied research to basic research and co-creational research. This group consisted of research officers (*n* = 2), training and research coordinators (*n* = 2), university lecturers (*n* = 2), directors of mental health services (*n* = 2), a research associate (*n* = 1) and a head of research department (*n* = 1). We ensured that young people (*n* = 2, aged under 25 from Indonesia and Romania) were included in the exercise to capture the voices of youth meaningfully (22) (See **Table 1**).

**Table 1:** Demographic and other information pertaining to the study participants.

Country	Gender	Age	Position	Scientific discipline	Any personal lived experience*
Latin America					
Colombia	Female	Above the age of 25	Lead Project coordinator	Psychology	No
Ecuador	Male	Above the age of 25	Minister of Public Health	Public Health	No
Africa					
Ghana	Female	Above the age of 25	University Lecturer	Psychology	No
Tanzania	Male	Above the age of 25	Senior Research and Programs Officer	Public Health	No
Senegal and Sierra Leone	Female	Above the age of 25	Research Associate	Public Health	No
Europe					
Romania	Female	Under the age of 25	Training and Research Department Coordinator	Psychology	Yes
Asia					
India	Female	Over the age of 25	Research Associate	Mental Health Law and Policy	No
Indonesia	Female	Under the age of 25	Community Mental Health Specialist & Youth Advisor	Psychology	Yes
Pakistan	Female	Above the age of 25	Director of Mental Health Services	Public Health	No
Vietnam	Male	Above the age of 25	Head of Research Department	Psychology	No

### Ethical considerations

The workshop took place in the form of a meeting, within an event room at a hotel in Nairobi, Kenya, and therefore, no ethical approval was obtained, but each representative provided verbal consent to have their data written into a report and a manuscript for publication (15). Specifically, our study embraces the reciprocal trust necessary between parties engaging in research (i.e., participants and the host), which was confirmed by consenting to participation. We based our approach in the theoretical paradigm conceived by Korsgaard and colleagues that draws on reciprocal trust (23, 24). This paradigm encapsulates three distinct dimensions that include: 1) trust spirals; 2) trust gained over time as the dynamic nature of relationships evolve and 3) trust trajectory. Therefore, it is in this theoretical underpinning that the principles of ethics were embedded in our study.

### Consensus-building process

The main aim of the NGT is to generate themes that are discussed and ranked by the group. In this regard, the NGT method involves an inclusive process designed to encourage equal and democratic participation (25, 26), which was used to gather and prioritize information on key stressors across geographical contexts. This mixed-methods approach identifies issues, allows for in-depth qualitative discussions, and allows for prioritizing or ranking key stressors (quantitative).

**Figure 1:**
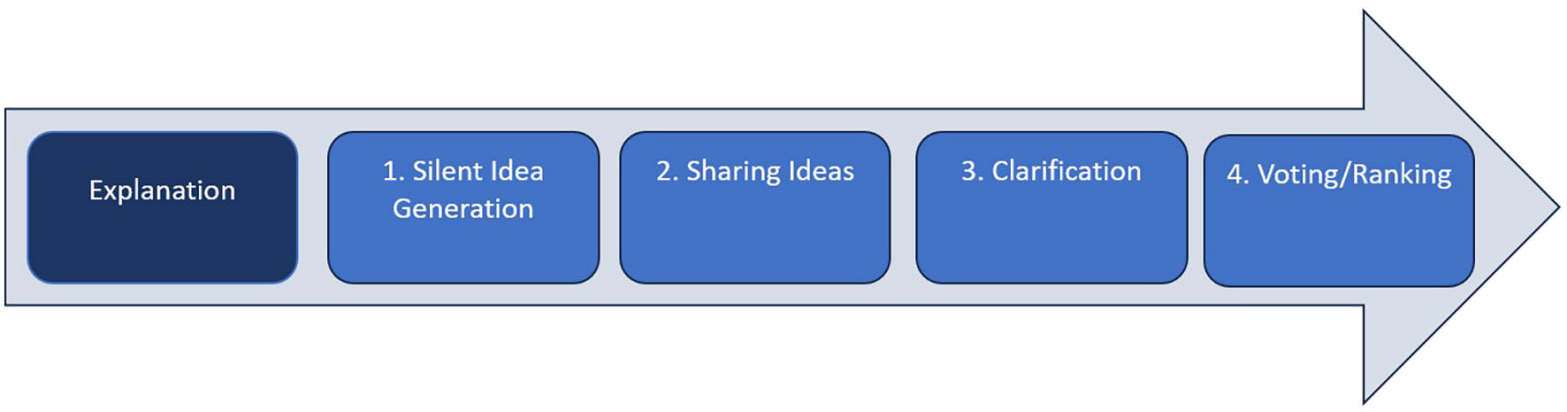
Research prioritised exercise.

The five steps involved in the NGT process (see **Figure 1**) include: 1) explanation; 2) silent individual generation of ideas; 3) recording of all representatives’ ideas (in a round-robin format); 4) group discussion of all generated ideas (to organize the list and remove duplications); and 5) voting on the priority of items.

**Figure 2:**
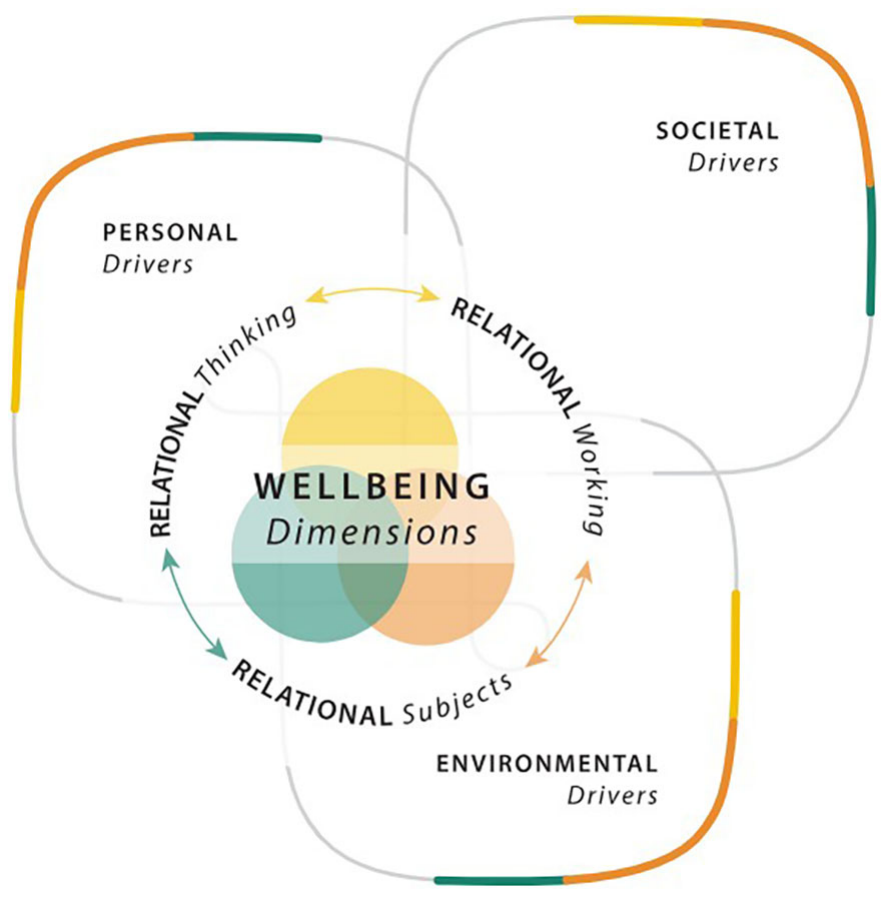
The relational wellbeing model.

By design, the NGT process enables the active engagement of all representatives. In this way, the outcomes of the NGT were not subject to interpretation by the facilitator (BB), nor were they dominated by the more vocal group members. An important benefit of the NGT process for this project was that it allowed for building consensus and did not require extra time for analysis (27). The NGT session was conducted in English and ran for approximately 120 minutes (see **Figure 1**). As a first step, the facilitator described the NGT as a method to the group, who then had the opportunity to ask questions. This introduction (or explanation stage lasted 5 minutes) and was a factual description of the method’s different steps, and did not have any content or comment that would influence participants and the task in hand. After the explanation stage, the facilitator asked the group the nominal question: “*What are the most pressing stressors related to youth mental health deserving of research, and how do we develop a richer understanding of the long-term effects of these stressors?*” The question was displayed on a PowerPoint slide, and all representatives were asked to respond to the question on post-it notes (silent idea generation which lasted 20 minutes) and were subsequently collected by the facilitator. It was emphasized to the group for them to avoid personal biases and that they had a responsibility to follow the line of inquiry set out in the nominal question. At this stage, the panel was given no guidance on how broad or narrow their focus should be. However, each representative was asked to apply the Relational Wellbeing (RWB) framework of Shreya Jha and Sarah White (see **Figure 2**), which was carefully explained to them during the session (21). In particular, there are five distinct ‘relational components’ to the RWB framework (i.e., R1: relational subject; R2: means through which needs are addressed; R3: inter-relations of personal, societal and environmental drivers of wellbeing; R4: conduits of power and making of identities; and R5: inter-relations of concepts and research methods with representations of wellbeing), and for this particular paper we focused on R3 where inter-relations between the experience of wellbeing and the underlying factors within persons and their contexts that either promote or undermine wellbeing predominate (20), an aspect that would allow us to identify and separate potential relations between stressors and mental health. We refer to these inter-relations as ‘drivers’ of wellbeing at the personal, societal, and environmental levels. Personal drivers include factors such as personality, personal history, personal endowments, and interactions with others in close relationships and within the community. Societal drivers are characterized by the practices of collective social arrangements through which societies are organized, generating differences among groups of people, institutional structures and processes, social forums, and culture. Environmental drivers recognize the interdependence between living beings and the earth, affecting wellbeing through air quality, biodiversity, and the built environment (20). The RWB framework was used as a guiding tool for the NGT, starting with an explanation of the three drivers, as described above. Following this, each representative wrote down what they felt based on their experience as key stressors related to youth mental health and placed sticky notes under the relevant drivers (i.e., personal, societal, and environmental)—viz, sharing ideas (40 minutes).

The facilitator then led an open discussion on each idea (i.e., stressor) to ensure that all representatives understood them. When multiple representatives suggested an idea or where the group recognized highly similar ideas, these ideas were combined to form a single ‘idea’.

In order to tackle power dynamics within the group, the facilitator actively encouraged participation from less vocal members. Consensus was defined as achieved when there were no further comments or suggestions for corrections from any participants (i.e., saturation). Notably, consensus determined the rigor of the data collected and secured communicative validity (28). In this context, no member had difficulty answering the question or presenting their views.

A rapporteur (NI) took notes to reflect the range of discussion on the research question, ensuring representatives’ anonymity. Following the discussion of each idea, the representatives independently ranked the ideas electronically. The resulting ranked idea represented the group’s prioritized responses to the question. The results were then shared with the group for clarification (60 minutes). For the voting step (20 minutes), representatives received an email with a link to anonymously vote and rank the stressors based on their experience working in youth mental health. Representatives voted according to: 1) the prevalence of the stressor within their countries; 2) whether addressing the stressor would be valuable; 3) the short and long-term impact of the stressor, and 4) whether the stressor is considered a national priority. It is essential to highlight that there were discussions about some of the stressors being cross-cutting in relation to the different drivers of the RWB framework (viz., not being assigned to a single driver). The ranking of these stressors was submitted anonymously using KoboToolBox (a collection of web applications that allows for the analysis and storage of data either online or offline) (29). The data were analyzed offline.

## Results

This section presents the results stemming from the NGT exercise that were calculated by scoring each stressor based on its rank across the three drivers of the RWB framework. Each stressor was ranked according to first and last choice, where stressors per domain were ranked according to aggregated scores from each stakeholder—based on subjective importance according to prevalence of the stressor in their country, whether tackling of the stressor be translated to value, short-term or long-term impact of the stressor and whether the stressor is considered a national priority—with the lowest scoring stressors achieving a higher rank than a higher scoring stressor. The list of stressors prioritized through representative ranking can be seen in **Table 2**.

**Table 2:** List of stressors according to rank.

Personal stressors ranked		Societal stressors ranked		Environmental stressors ranked	
Mental health awareness	1	Stigma	1	Media	1
Mental health knowledge/literacy	2	Violence and conflicts	2	Policy environment	2
Psycho-social/socio-emotional issues	3	Access to mental health systems	3	Technology	3
Education expectations (Academic pursuit)	4	Education expectations (Parental pressure, peer pressure and socio-cultural pressure)	4	Climate change	4
Substance abuse	5	Negative school environment	5	Food insecurity	5
Poverty	6	Economic inequality	6		
Childhood trauma	7	Policy environments	7		
Food insecurity	8	Bullying	8		
		Food insecurity	9		

### Personal stressors

Across the 11 countries, representatives ranked poor mental health awareness as a key personal stressor. This was linked to the limited understanding and awareness of adolescent mental health and wellness in these countries. Relatedly, poor mental health knowledge and literacy were ranked second. Representatives discussed that mental health literacy extends beyond merely knowing the definitions of mental health conditions. It involves recognizing symptoms and warning signs, understanding associated life challenges, and knowing where to find support within one’s country. Other stressors that stood out at the personal level included poverty, childhood trauma, substance abuse and educational expectations.

### Societal stressors

Stigma in healthcare environments, which can impact individuals’ mental health and wellbeing, was ranked as the highest societal stressor. This includes negative attitudes by healthcare providers towards patients with mental health problems. Such stigma can contribute to inadequate care and reluctance to seek treatment—by youth—because of the shame and guilt that may come along with admitting that one is struggling with a mental health condition. Beyond stigma, aspects of violence and conflicts in addition to bullying and a negative school environment, were listed among key stressors likely to affect youth mental health at the societal level.

### Environmental stressors

In terms of environmental stressors, media, including social media and the digital world (e.g., gaming), was ranked as the highest stressor. Climate change and technology (e.g., the artificial intelligence revolution) were also identified as important stressors at the environmental level. Notably, the policy environment was highlighted as an important stressor in relation to youth mental health at both the societal and environmental levels.

### Food insecurity as a cross-cutting stressor

Although food insecurity was ranked as the stressor least likely to impinge on youth mental health across the three categories, it remains a crucial factor to wellbeing. In LMICs, some youth may be unemployed or live in households where parents or guardians have limited financial resources for basic necessities, including food. This results in food insecurity, which leads to stress, anxiety, and uncertainty about meeting basic needs, which in turn, can place a toll on mental health.

## Discussion

The results of the present study reveal that a diverse multi-stakeholder group stemming from 4 continents successfully reached consensus on 18 distinct psychological stressors that impact mental wellbeing among youth (see **Table 2**). These stressors are related to the personal, social, and environmental drivers of the RWB framework, focusing particularly on youth mental health.

Children and young people in LMICs are vulnerable to developing mental disorders (30). Studies have found that fewer children and young people in LMICs seek professional help compared to HICs (31), due to a lack of mental health awareness. In our workshop activity, mental health awareness and mental health literacy were found to be the main stressors within the personal category. This reflects findings of a recent study which identified a more consistent pattern of poor knowledge and low levels of awareness of mental health problems among children and young people in LMICs (31). Low levels of mental health awareness were associated with pervasive stigma towards those with mental illness (31). Notably, mental health awareness extends to the reality that many people struggle to differentiate between the role of psychiatrists, psychologists, and social workers in the mental health field. In this regard, extant literature reveals an association between low mental health literacy among adolescents in LMICs in terms of help-seeking and effective treatment received (31). Mental health literacy is defined as an individual’s knowledge and beliefs about mental disorders that assist their recognition, management, or prevention (32). A study conducted by Renwick et al. (31) found that knowledge of mental illnesses, treatments, and help-seeking among populations in studies conducted in LMICs was poor, as well as the recognition of specific mental illnesses. It is, therefore, important to target mental health literacy to improve the health and wellbeing of younger populations. Much of what we know about mental health literacy is focused on HICs, and this is of particular concern given that the burden of mental health disorders is higher in LMICs, where access to mental health resources remains scarce (33). In this context, community interventions (e.g., the Friendship bench) are considered useful (31), as they target the individual’s mental health awareness and knowledge through problem solving and educational aspects.

Stigma was identified as one of the top social stressors across country settings. This corroborates the extant literature stating that the stigma of mental health manifests at the societal level (34). The stigma that is associated with mental health results in delayed help-seeking, reduces individuals’ willingness to access health services, and increases the risk of individual human rights violations (34). Stigma was related to mental health awareness under the personal stressor category because there are certain attitudes and (cultural) beliefs held towards mental illness and treatment. However, several studies concluded that there is inconsistent evidence of the interrelationship between mental health knowledge and stigma (31). Evidence from a meta-analysis revealed that stigmatizing attitudes that others hold towards people with mental illness play a role in them seeking treatment for mental health problems (31). Stigma levels are closely linked with mental health challenges faced by children and young people in LMICs (31). A range of solutions have been developed to address mental health stigma in LMICs. These solutions include developing comprehensive and inclusive legislation, developing mental health programs, collaborating with schools and other institutions to educate youth on mental health literacy (e.g., the culturally adaptive Anansi programme developed by the Shamiri Institute that targets the mental health needs of low-income youth in Kenya), and integrating mental health services into the health care system to allow children and young people access to mental health care (34). To this end, the African Union suggests that we should implement mental health awareness campaigns and add mental health education to the school curricula with the aim of reducing stigma and increasing young people’s knowledge on the topic (35). Considering the influence of stigma on help-seeking, many LMICs have declared that implementing anti-stigma interventions must be a priority for health policy (36). This aligns with why policy was listed as a main stressor among the list of environmental stressors.

In this context, Zhou and colleagues reveal that policy focusing on child and adolescent mental health is important for the development of mental health systems targeting this vulnerable stratum of society (37). They argue that there is a universal lack of policy focusing on child and adolescent mental health, especially in LMICs (37). Consistent with this, a survey conducted by Shatkin and Belfer (5) found that only 18% of countries (35 of 191) interrogated had mental health policies, which might have some beneficial impact on children and adolescents (37). The WHO Child Mental Health Atlas published in 2005 also demonstrated a paucity of child and adolescent mental health policies, as only 30% of the 66 reported countries had national child and adolescent mental health policies (38). This shows the need to raise awareness and provide local experience and expertise for formulating, implementing, and promoting child and adolescent mental health policy in LMICs. Therefore, it is crucial to develop and strengthen policies and legal frameworks that prioritize young people’s mental health, including protection from online spaces and cyberbullying, and which promote destigmatization (35). Additionally, policy-makers should involve young people in the policy-making process to create more targeted and effective policies (35). Notably, the African Youth Charter advocates for youth participation in policies and programmes at local, national, regional, and international levels as an important form of youth engagement and as a means of peer-to-peer training (39).

Access to media (e.g., social media) was ranked as one of the top environmental stressors. The application of technology in healthcare is rapidly increasing, with some research suggesting the potential benefit of digital mental health technologies in LMIC settings (15), although the negative effects on mental health cannot be overlooked (e.g., cyber bullying and addiction). In this regard, a study conducted by Carter and colleagues revealed that there have been improvements in the design and implementation of digital mental health interventions in LMICs, as well as the application of more rigorous research methods as the field continues to evolve (40). Most of the world’s social media users are situated in LMICs, which means that there are opportunities to leverage this aspect and create platforms to support mental health promotion efforts and service delivery (41). This indicates that there are prospects for online interventions that focus on mental health in LMICs. In this context, the African Union draws attention to the need for resources to be allocated to mental health services and awareness campaigns geared towards young people so as to address the mental health challenges that these individuals experience in digital spaces (35).

The stressors that appeared at the top of the three drivers of the RWB framework, showed that adolescents’ stress can be conceptualized as stress related to mental functions, attitudes, and relationships, which is placed on them by their parents, school, and society. However, many adolescents in LMICs feel a great level of pressure due to an increase in social and economic responsibilities, resulting in them seeking employment, which limits their higher education prospects (42). This speaks to those stressors found among the midst of the ranked stressors (see **Table 2**), such as violence and conflicts, access to mental health systems, economic inequality, education expectations, and negative school environment. For example, there may be pressure at school for adolescents to do well, conflicts with teachers or peers, and domestic disputes. Although schools can provide a potential location to address the mental health needs of the child, Fazel et al. (42) emphasize that schools in LMICs offer few opportunities for educational development due to the challenges of teaching in low-resource contexts. Parents also play an important role in assisting youth with the demands related to school and social relationships that promote wellbeing and in the process prevent stress among children and young people (11). However, a high number of children in LMICs face various risks throughout their life, including an absence of caregivers, problems with physical health, and nutritional status (42).

Food insecurity was ranked last among personal, societal, and environmental drivers, despite this issue remaining a significant problem among people living in LMICs (43), particularly as one considers climate change. According to the African Union, young people are affected by the climate crisis, rendering them particularly vulnerable to psychological impacts. The direct effects of extreme weather events such as floods, droughts, and other climate-related disasters on the mental health of Africa’s youth are multifaceted (35), with research consistently revealing that individuals living in areas affected by these events experience both physical and mental health challenges (35). Food insecurity has been described as the limited availability of nutritionally adequate and safe foods or the limited ability to acquire acceptable foods in a socially acceptable way (44). It was found that long-term hunger can have psychological effects on individuals. More specifically, a study conducted by Ae-Ngibise et al. (44) in LMICs reported that exposure to food insecurity is associated with increased psychological distress, worries, anxiety, sleep loss, and intellectual disability, and these findings are broadly consistent with literature from HICs. Similarly, Smith and colleagues assert that food insecurity may be a risk factor for depression in adolescents, and their study findings showed that there was a high prevalence of depressive symptoms and food insecurity among adolescents in LMICs (45). Further, these authors reveal some reasons as to why these two aspects correlate, including: 1) food insecurity can induce feelings of stigma due to material deprivation and increase the risk of depression; 2) food insecurity is associated with poorer educational outcomes as it may act as a psychological or emotional stressor affecting behavior; 3) and food insecurity results in higher levels of stress that may lead to high levels of depression (45).

Limitations of the current study include that the group consisted of 10 participants, which did not capture information from the entire 12 countries encapsulating the Being initiative (viz., we missed input from Morocco). This may have proven of significance from a cultural aspect, given that the predominant religion in Morocco is Islam, and how this may feed into the stressors that adolescents and young people face in this country (46). Just as important is that the main composition of the participants in this study was mental health researchers. And while it is widely appreciated that a variety of stakeholders (e.g., policy makers, advocacy group, family members and care givers) tends to provide wider perspectives, generate better knowledge outcomes as well as create a greater sense of ownership, in this case we placed particular emphasis on engaging with purposefully selected individuals with research experience in the mental health space given the specificity of the nominal research question. To this end, we acknowledge that the current study sample was small and that a selective sample can undermine validity by introducing bias and increasing error, limiting the accuracy of results. This impacts generalizability by failing to represent the larger population. To mitigate these effects we encourage future studies to include larger sample sizes and to implement random sampling techniques as a means to minimize selection bias.

Another limitation of the study was the preponderance of women. A balanced ratio would enhance perspectives on gender-specific stressors. Specifically collecting balanced data on individuals across gender would enable more accurate conclusions to be drawn on the influence of gender in relation to stressors that affect youth mental health. Although the exercise included two young people (both female aged under 25) with lived experiences of mental health challenges, capturing the voices of additional young people (from other geographic regions) would have strengthened the relevance and significance of the data that was collected (22). Lastly, we did not record the conversations that took place during the NGT exercise, which limited the ‘richness’ of information (i.e., potential themes) that could have been captured using a more rigorous qualitative approach (viz., coding). For instance, understanding why specific stressors were ranked accordingly and how they may have been important to particular countries and cultural contexts. Even so, our study does provide a starting point for further research and should be used as such.

## Conclusion

The NGT is a swift and effective method to derive a consensus of ideas or values. This study employed the NGT so as to gain a cross-national understanding of the stressors that impinge on youth mental health. Because the findings from the present study represent a selective perspective from a small group, it follows that further research be conducted regarding the diverse effects of emerging stressors on young people’s mental wellbeing. Specifically, stronger empirical work (e.g., multi-country Delphi panels, surveys, or mixed-methods studies involving youth directly) is needed to validate and expand on the stressors that were identified.

The findings from the present study revealed that although the representatives came from diverse countries and professional backgrounds, they all agreed on the list of stressors and how they impact young people’s mental health in an LMIC context. An increasing level of the published literature has identified potential solutions that can be implemented to assist youth who may be experiencing mental health challenges, but this depends on the resources available within the different countries. Further research is therefore required to identify possible solutions and strategies that can be utilized to create awareness and educate youth when they are faced with mental health challenges. It is also recommended that researchers explore the different interventions that can be applied to assist youth with mental health problems in LMICs. Lastly, more research should focus on knowledge gaps associated with the identified stressors (e.g., climate change) in terms of youth mental health, which can then better inform discourse around funding, mental health policy and practice.

## Data Availability

The raw data supporting the conclusions of this article will be made available by the authors, without undue reservation.
